# OPG/RANK/RANKL axis relation to cardiac iron-overload in children with transfusion-dependent thalassemia

**DOI:** 10.1038/s41598-023-39596-3

**Published:** 2023-08-02

**Authors:** Samira Zein Sayed, Asmaa Hosni Abd El-Hafez, Mostafa Ahmed Abu El-ela, Mohamed Aboul-fotouh Mourad, Suzan Omar Mousa

**Affiliations:** 1 Department of Pediatrics, Faculty of Medicine, Minia University, El Minya, Egypt; 2 Department of Clinical Pathology, Faculty of Medicine, Minia University, El Minya, Egypt; 3 Department of Radiodiagnosis, Faculty of Medicine, Minia University, El Minya, Egypt

**Keywords:** Biomarkers, Diseases, Risk factors

## Abstract

OPG/RANK/RANKL axis was reportedly involved in initiating various diseases, especially bone and cardiovascular diseases. This study aimed to assess the relationship between some OPG, RANK, and RANKL polymorphisms and alleles and iron-overload-induced cardiomyopathy in children with transfusion-dependent thalassemia (TDT). This study included 80 TDT children and 80 age and sex-matched controls. Real-time PCR was done for rs207318 polymorphism for the OPG gene and rs1805034, rs1245811, and rs75404003 polymorphisms for the RANK gene, and rs9594782 and rs2277438 polymorphisms for the RANKL gene. Cardiac T2* MRI and ejection fraction (EF) were done to assess the myocardial iron status and cardiac function. In this study, there were no significant differences in frequencies of the studied polymorphisms between cases and controls (p > 0.05 in all). In TDT children, OPG rs2073618 (G > C) had a significant relation to myocardial iron overload (p = 0.02). Its C allele had significantly more frequent normal EF than its G allele (p = 0.04). RANK rs75404403 (C > DEL) had a significant relation to cardiac dysfunction (p = 0.02). Moreover, the C allele of that gene had significantly more frequent affected EF than its DEL allele (p = 0.02). The A allele of RANKL rs2277438 (G > A) had significantly less frequent severe cardiac iron overload than the G allele (p = 0.04). In conclusion, the OPG/ RANK/RANKL genes may act as genetic markers for iron-induced cardiomyopathy in TDT children. Some of the studied genes’ polymorphisms and alleles were significantly related to myocardial iron overload and cardiac dysfunction in TDT children.

## Introduction

Beta thalassemia syndromes are a group of hereditary disorders characterized by genetic mutations resulting in reduced or absent beta-globin chains. The clinical severity of beta-thalassemia ranges from severe transfusion-dependent anemia in the homozygous state to mild to moderate microcytic anemia in the heterozygous state, depending on the severity of the beta-globin gene mutation and co-inheritance of other genetic determinants^1^. It has been estimated that one thousand children out of 1.5 million live births are born each year suffering from thalassemia in Egypt. It is reported that the carrier rate in Egypt is between 9 and 10% of the population^2^

The most critical morbidities in thalassemia are related to iron overload resulting from multiple blood transfusions and enhanced intestinal iron absorption^3^. After transferrin binding sites are saturated, non–transferrin-bound iron (NTBI) is transported through Ca^+2^ channels into hepatocytes, cardiac myocytes, and endocrine glands. Reactive oxygen species produced by the metabolism of NTBI contribute to cellular dysfunction and apoptosis^1^.

When iron starts to accumulate in the liver, the hepatic function remains normal or is slightly affected early in the disease. Patients with thalassemia often develop finely nodular hepatic cirrhosis after several decades^4^. In addition, iron accumulates in cardiac myocytes, particularly the ventricular walls, which causes left ventricular diastolic dysfunction, subsequent pulmonary hypertension, and eventually right ventricular dilatation and heart failure^5,6^.

While osteoprotegerin (OPG) is a cytokine of the tumor necrosis factor (TNF) receptor superfamily, receptor activator of nuclear factor kappa-B (RANK)/receptor activator of nuclear factor kappa-B ligand (RANKL) is a receptor-ligand pair of the TNF receptor superfamily. The OPG/RANK/RANKL system is considered the key molecular pathway in bone metabolism^7^.

The OPG/RANK/RANKL axis was discovered in the late 1990s. Its effect on immunity and dendritic cells and its role in bone homeostasis was verified. Recent studies revealed the contribution of the OPG/RANK/RANKL system to the emergent field of osteoimmunology, organogenesis, and disease conditions including, cancer and rheumatoid arthritis^8^.

Moreover, studies had linked the OPG/RANK/RANKL axis to many hepatic diseases such as non-alcoholic fatty liver disease^9^, chronic alcoholic liver disease^10^, and primary biliary cholangitis^10^, and other studies had linked it to many cardiovascular diseases^12–15^.

We found that the OPG/RANK/RANKL axis is linked to cognitive impairment in children with transfusion-dependent thalassemia (TDT)^16^.

In this study, we wanted to explore the relationship between genetic variants of OPG, RANK, and RANKL and iron-overload-induced cardiac disease in children with TDT.

## Subjects and methods

### Study design and participants

This prospective cross-sectional study was conducted at the pediatric department, of Minia University Children and Maternity Hospital, from September 2019 to March 2022.

We analyzed the data of the 80 TDT children who regularly come to follow-up visits in the pediatric hematology clinic at Minia University Children’s Hospital. We also included 80 healthy children as a control group who were age and sex-matched with the TDT children. The study was explained in detail to the parents or legal guardians of the participant children and written informed consents were taken from them. The study was designed respecting the expected ethical aspects. It was performed according to the Declaration of Helsinki 1975, as revised in 2008 and approved by the Institutional Review Board and Medical Ethics Committee of Minia University.

Included children were on regular blood transfusion programs (transfusion-dependent). All patients received repeated blood transfusions (10 ml packed RBCs /kg body weight) every 2–6 weeks to keep their hemoglobin level (Hb) around 9 g/dl after each transfusion. All patients were on deferasirox therapy for at least 12 months before being recruited into the study. Their age ranged between 5 and 16 years, with no sex predilection.

We excluded from our study TDT children who had a history of congenital or acquired heart disease, a history of cardiac surgery, were on cardioprotective drugs or iron chelation therapy other than deferasirox.

### Sample calculation

Fisher’s formula for sample size determination was used; n = z^2^Pq/d^2^. Where n = desired sample size population; Z = standard normal deviation—set at 1.96 at 95% confidence level. P = proportion of the subjects that present the characteristic. For this study, P will be estimated at 0.5, q = 1 − P. Therefore, the desired sample size was calculated to be n = 42.68. When considering the 20% dropout of study participants, so, the required sample size will be 52.

### Baseline clinical assessment

All included children were subjected to detailed medical history taking and thorough clinical examination. We emphasize in children with TDT on the history of the age of their first transfusion, transfusion burden/year (ml/kg/year), history of splenectomy, the average frequency of transfusion, and type and duration of chelation therapy.

### Laboratory investigations

#### Sample collection and biochemical analysis

Six ml of venous blood was withdrawn from each subject by aseptic venipuncture. This sample was divided as follows: Two ml were collected on two vacutainer tubes containing EDTA solutions, one tube was used for CBC assay, by automated cell counter (CelltacES, Nihon Kohden, Germany), and the other for DNA extraction. The other four ml of venous blood were put in a serum separator gel tube, and it was allowed to clot for 30 min at 37 °C before centrifugation for 15 min at 3,500 rpm. The separated serum was used for measurement of serum ferritin and liver function tests, and the remaining serum was stored at −20 °C for further assessment of additional investigations. Liver function tests were assayed using fully automated clinical chemistry auto-analyzer system Konelab 60i (Thermo Electron Incorporation, Finland). Ferritin was assayed by indiko clinical chemistry system, Thermo, Finland.

#### Molecular analysis

Real-time PCR was done for the following SNPs: rs 9594782 **(C > T)**, 2277438 **(G > A)** for RANKL polymorphisms, rs 1805034 **(C > T)**, 1245811 **(A > G)**, and 75404003 **(C > DEL)** for RANK polymorphisms, and rs 207318 **(G > C)** for OPG polymorphism. It was carried out on DT lite 4 Real-Time PCR System (DNA Technology, Russian) using the following program: one cycle of incubation at 50 °C for 2 min then one cycle for 10 min at 95 °C and lastly 40 cycles of incubation at 95 °C fo 00:15 and then for 1:00 min Results were interpreted on software DT lite 4 7.9

### MRI

Liver iron concentration (LIC) and T2* MRI were performed in the Department of Radiology, Minia University Children and Maternity Hospital, using MR Philips ingenia 1.5 Tesla (Philips Medical Systems, Netherlands), ECG & respiratory-gated with dedicated phased array Torso coil using single breath-hold multi-echo gradient echo sequence.

Regarding cardiac MRI (CMRI), the acquired images were post-processed using Region-Based Measurement to calculate: myocardial T2* value: a single short-axis mid-ventricular slice was acquired using a single breath-hold ECG-gated multi-echo dark blood technique. This T2* sequence generated a series of eight images with TEs of 1.5–17.3 ms and spacing of 2.3 ms., then a region of interest (ROI) is put in each image to measure signal intensity. For measurement in the heart, correction for positional changes between breath-holds was made to ensure regions of interest were within the myocardium. In addition,—left ventricular ejection fraction (EF) was measured using standard CMR sequence, and EF < 56% was considered an indicator of cardiac dysfunction^17^.

Myocardial T2* decay was calculated using manual analysis in an electronic spreadsheet with semi-automated analysis software using Thalassemia tools (a plug-in of CMR tools, Cardiovascular imaging solutions, London, UK).

Results of cardiac T2* were categorized as severe (< 10 ms), moderate (10–14 ms), mild (14–20 ms), and acceptable (> 20 ms) myocardial involvement^18,19^.

### Statistical analysis

Data will be coded, entered, and analyzed using SPSS (statistical package for social sciences) version 20. descriptive statistics were calculated and expressed as (mean ± standard deviation (SD), range, median and interquartile range (IQR)). The quantitative variables will be compared using paired t-test or one-way ANOVA. The comparison of qualitative variables will be performed using chi-square test or Fisher’s exact test. p-value < 0.05 was considered statistically significant.

## Results

The demographic, clinical and basic laboratory data of the TDT children and controls are represented in Table 1.

**Table 1:** Demographic, clinical and laboratory data of TDT children and control.

Variables	TDT children (n = 80)	Control (n = 80)	p-value
Age (years): Mean ± SD	13.2 ± 3.9	13.3 ± 4.6	0.7
Sex: Male: n (%)	49(61.3%)	46(53.5%)	0.4
Consanguinity: Positive: n (%)	48(60%)	40(50%)	0.8
BMI: Mean ± SD	17.9 ± 3.1	20.3 ± 15.4	0.04*
Laboratory investigations:			
Hb (gm%): Mean ± SD	9.2 ± 0.7	11.1 ± 0.9	< 0.001*
ALT (iu/l): Mean ± SD	80.2 ± 62	19.03 ± 4.5	< 0.001*
AST (iu/l): Mean ± SD	69.4 ± 53.4	22.5 ± 5.3	< 0.001*
Ferritin (ng/ml): Mean ± SD	4975.6 ± 2903	34.2 ± 13	< 0.001*
Age of first transfusion (months): Mean ± SD	18.7 ± 16	–	–
Pre transfusion Hb (gm%): Mean ± SD	6.1 ± 0.51	–	–
Splenectomy: n (%)	39(48.8%)	–	–
Age of starting chelation therapy (years): Mean ± SD	6.23 ± 5.2	–	–
Liver iron concentration (LIC): mg/g dry weight	13 ± 7.2		

BMI: body mass index; Hb: hemoglobin; ALT: alanine transaminase; AST: aspartate transaminase.

Significant values are in [bold].

**Figure 1:**
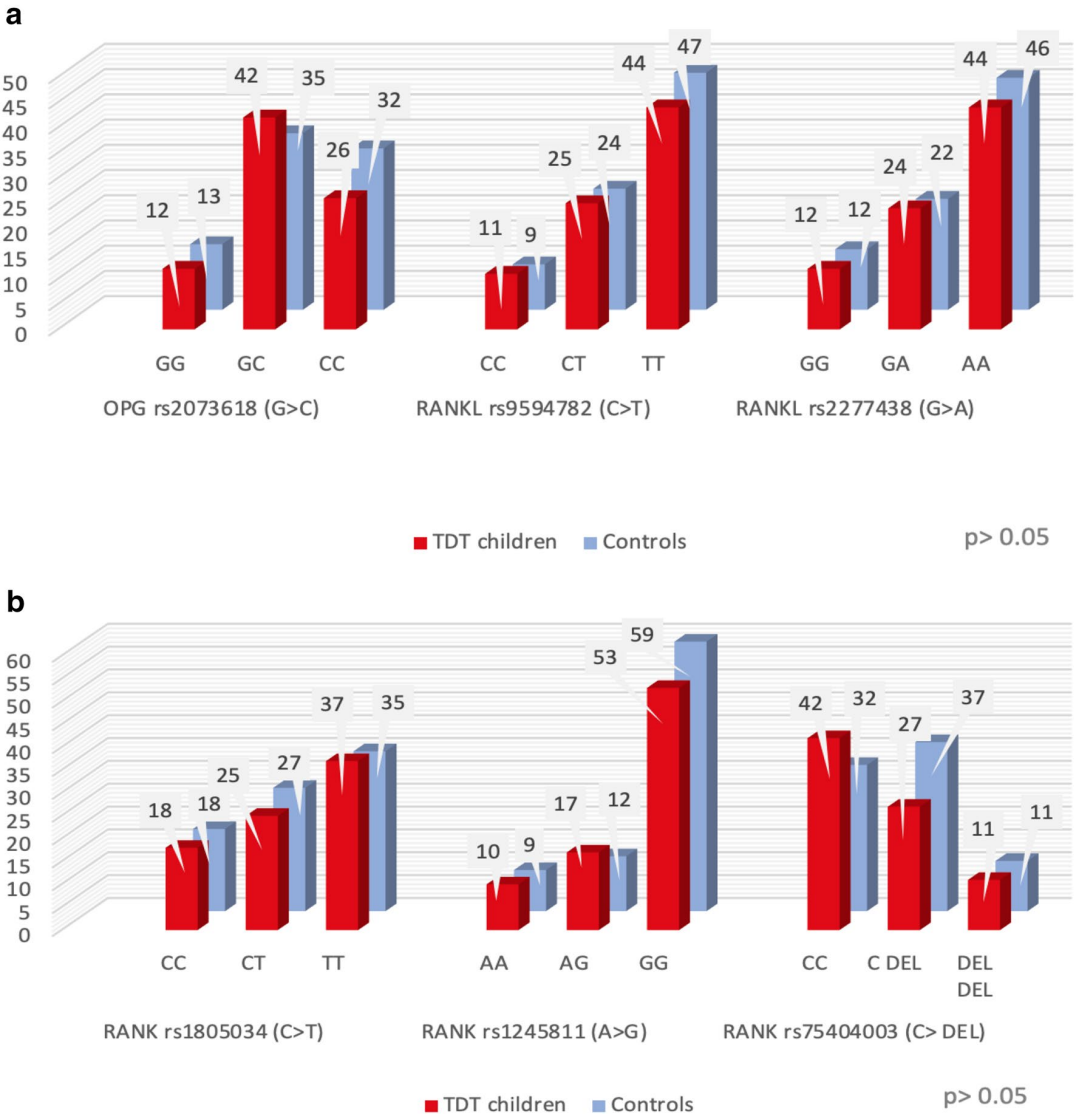
(**a**) and (**b**) Frequency distribution of the different polymorphisms of the studied genes within the two studied groups.

The studied polymorphisms genotypes in patients were not significantly different from those of the controls. (Fig. 1a,b).

TDT children had significantly lower T2 and EF than controls, as p < 0.001in all (Table 2).

**Table 2:** Comparison between MRI results in TDT children and control.

Variable	TDT children N = 60 Mean ± SD	Control Group N = 60 Mean ± SD	p-value
T2*(ms):	17.21 ± 7.25	28.8 ± 5.3	< 0.001*
EF (%):	57.6 ± 6.9	68.2 ± 6.2	< 0.001*

LIC: Liver iron concentration; T2*: T2 sequence of MRI; EF: Ejection fraction.

*Statistical significance < 0.05.

Significant values are in [bold].

26 (32.5%) TDT children were categorized as having severe myocardial iron-overload (T2* < 10 ms), while 32 (40%) TDT children were considered as having myocardial dysfunction with an EF < 56%.

OPG rs2073618 (G > C) had a significant relation to myocardial iron overload in the TDT children measured by T2* (p = 0.02, OR = 0.06, 95% CI 0.01–0.67). TDT children with its GG polymorphism were significantly more frequent to have severe iron-overload myocardial involvement than the GC polymorphisms (p = 0.02). Moreover, the A allele of RANKL rs2277438 (G > A) had significantly less frequent T2* < 10 ms than the G allele (p = 0.04, OR = 0.9, 95% CI 0.8–0.94) (Table 3).

**Table 3:** Genotype and allele frequency in TDT children according to cardiac T2* MRI.

Genotype			Cardiac T2 *		p	OR (95% CI)
			Non-severe (n = 54)	Severe (n = 26)		
OPG rs2073618 (G > C)	Polymorphism	GG	2	10	0.02*	REF
		GC	34	8		0.06 (0.01–0.67)
		CC	18	8		0.1 (0.01–1.3)
	allele	G	38	28	0.3	REF
		C	70	24		1.8 (0.6–6.1)
RANK rs1805034 (C > T)	Polymorphism	CC	12	6	0.1	REF
		CT	14	11		0.4 (0.1–1.6)
		TT	28	9		0.1 (0.04–1.5)
	allele	C	38	23	0.9	REF
		T	70	29		1.1 (0.3–3.8)
RANK rs1245811 (A > G)	Polymorphism	AA	3	7	0.6	REF
		AG	9	8		0.3 (0.02–7.1)
		GG	42	11		0.3 (0.02–3.4)
	allele	A	15	22		REF
		G	93	30		0.5 (0.1–1.1)
RANK rs75404003 (C > DEL)	Polymorphism	CC	30	12	0.5	REF
		C DEL	20	7		0.4 (0.04–3.6)
		DEL DEL	4	7		1.9 (0.2–21)
	allele	C	80	31	0.9	REF
		DEL	28	21		0.95 (0.24–3.8)
RANKL rs9494782 (C > T)	Polymorphism	CC	5	6	0.7	REF
		CT	16	9		0.2 (0.1–3.4)
		TT	33	11		1.9 (1.5–2.8)
	allele	C	26	21	0.6	REF
		T	82	31		1.6 (0.3–7.7)
RANKL rs2277438 (G > A)	Polymorphism	GG	6	6	0.13	REF
		GA	17	7		1.0 (0.5–2.1)
		AA	31	13		0.03 (0.01–0.2)
	allele	G	29	19	0.04*	REF
		A	79	33		0.9 (0.8–0.94)
Post hoc analysis						
OPG rs2073618 (G > C)	GG vs GC	GC vs CC	**GG vs CC**			
	0.02*	0.07	0.08			

*Statistical significance < 0.05.

Significant values are in [bold].

RANK rs75404403 (C > DEL) had a significant relation to cardiac dysfunction in TDT children measured by ejection fraction (p = 0.02, OR = 6.1, 95% CI 1.7–22) and children with its CC polymorphism had a significantly more frequent cardiac dysfunction than its C DEL and DEL DEL polymorphisms (p = 0.02 and 0.01 respectively). Moreover, the C allele of that gene had a significantly more frequent impaired ejection fraction than its DEL allele (p = 0.02, OR = 2.8, 95% CI 1.1–6.9). Also, the C allele of the OPG rs2073618 (G > C) gene had a significantly more frequent normal ejection fraction than its G allele (p = 0.04, OR = 2.1, 95% CI 1.0–4.7) (Table 4).

**Table 4:** Genotype and allele frequency in TDT children according to cardiac dysfunction by EF.

Genotype			EF		p	OR (95% CI)
			Normal (n = 48)	Affected (n = 32)		
OPG rs2073618 (G > C)	Polymorphism	GG	7	5	0.06	REF
		GC	26	16		0.03 (0.01–0.9)
		CC	15	11		0.4 (0.2–3.4)
	Allele	G	40	26	0.04*	REF
		C	56	38		2.1(1.0–4.7)
RANK rs1805034 (C > T)	Polymorphism	CC	10	8	0.09	REF
		CT	19	6		3.5 (0.7–7)
		TT	19	18		0.9 (0.2–3.3)
	Allele	C	39	22	0.4	REF
		T	57	42		0.7 (0.3–1.5)
RANK rs1245811 (A > G)	Polymorphism	AA	5	5	0.34	REF
		AG	10	7		3.0 (0.2–4)
		GG	33	20		4.7 (0.45–8)
	Allele	A	20	17	0.2	REF
		G	76	47		2.34 (0.8–6.5)
RANK rs75404003 (C > DEL)	Polymorphism	CC	18	24	0.02*	REF
		C DEL	23	4		6.1 (1.7–9)
		DEL DEL	7	4		2.14 (0.3–4.5)
	Allele	C	59	52	0.02*	REF
		DEL	37	12		2.8 (1.1–6.9)
RANKL rs9494782 (C > T)	Polymorphism	CC	7	4	0.5	REF
		CT	13	12		0.5 (0.07–4)
		TT	28	16		1.1 (0.2–7)
	Allele	C	27	20	0.42	REF
		T	69	44		1.4 (0.6–3.3)
RANKL rs2277438 (G > A)	Polymorphism	GG	6	6	0.23	REF
		GA	13	11		1.8 (0.3–4.5)
		AA	29	15		3.7 (0.6–6.9)
	Allele	G	25	23	0.1	REF
		G	71	41		0.4 (0.2–1)
Post hoc analysis						
RANK rs75404403 (C > DEL)	**CC vs C DEL**	**C DEL vs DEL DEL**	**CC vs DEL DEL**			
	0.02*	0.4	0.01*			

EF: Ejection fraction.

*Statistical significance < 0.05.

Significant values are in [bold].

## Discussion

Iron overload-induced myocardial dysfunction is TDT's leading cause of morbidity and mortality^20^. In this study, TDT children had significantly lower T2* and EF than controls. Previous studies proved that decreased T2* values correlated with myocardial iron overload, deterioration of cardiac functions, and cardiac events in TDT patients^21–23^.

This study aimed to explore the relationship between genetic variants of the OPG/RANK/RANKL axis and iron-overload-induced cardiac abnormalities in children with TDT, as this axis was linked before to oxidative stress-induced disease^7^, and oxidative stress is the primary mechanism involved in thalassemia iron-overload-induced cardiomyopathy^24^.

Our study revealed that the OPG rs2073618 (G > C) genotype in TDT children had significant relation with myocardial iron measured by cardiac T2*, and children with its GG polymorphism had significantly more frequent lower T2* than the GC polymorphism. At the same time, the C allele of this gene had a significantly more frequently normal ejection fraction than its G allele.

Our study also found that the A allele of RANKL rs2277438 (G > A) gene had significantly less frequent lower T2* than the G allele. RANK rs75404003 (C > DEL) gene had a significant relation to cardiac function in TDT patients as its CC polymorphism had significantly more frequent cardiac dysfunction than its other two variants. Moreover, the C allele of that gene had a significantly more frequently affected ejection fraction than the DEL allele.

Following our findings, many studies demonstrated the relation of different polymorphisms of this axis with cardiac diseases*;* Singh et al. study determined that thalassemia patients having RANK rs75404003 (C > DEL), OPG rs2073618 (G > C), and minor C allele of OPG rs2073618, were at high risk for developing left ventricular hypertrophy^25^*.* A meta-analysis done by Song et al. in 2016 showed that the OPG rs2073618 genotype is related to cardiovascular disorders such as left ventricle hypertrophy, carotid plaques, and increased risk of stroke^26^. Also, Straface et al. reported that the CC polymorphism and the C allele of OPG rs2073618 were associated with unstable atherosclerotic plaques^27^.

Not only was this axis linked to cardiac diseases, but also it was linked to other systemic diseases. RANKL rs2277438 (G > A) had a significant relation to cardiac T2* in our study; other studies had found it to affect bone diseases. Abdi et al. found that its heterozygous GA polymorphism was associated with lower 25(OH) D in postmenopausal Saudi Arabian women^28^, and Rhee et al. showed its role in vascular calcification and bone metabolism^29^. The effect of the RANKL rs2277438 (G > A) genotype on both cardiac and bone disease can be explained by an intricate connection between osteogenesis and angiogenesis^30^. Studies revealed that shear stress and osteoclastic differentiation induce cardiac injury through the expression of OPG/ RANK/RANKL axis genes, in addition to their role in vascular calcification, which is a well-known risk factor for cardiovascular diseases^31–33^.

Iron overload induces free iron radicals, increasing oxidative stress in the myocardium^24^. The OPG/ RANK/RANKL axis is involved in the endothelial integrity of cardiomyocytes^7^. and is related to fibroblast growth factors (FGFs), which protect against oxidative stress-related endothelial damage^34^. Moreover, OPG, RANK, and RANKL genes were proven to have an active role in pathological angiogenesis, inflammation, and cell survival through Vascular endothelial growth factor^35^. Additionally, studies supported that this axis affects the activity of human fibroblast matrix metalloproteinase (MMP9), which directly relates to myocardial function under pathological conditions^36^. Confirming the previous studies, Rochette et al*.* reported that OPG level has a positive association with increased cardiovascular risk and suggested that the increase in OPG levels represents a protective mechanism in response to vascular damage. They concluded that circulating OPG levels can be used as independent prognostic biomarkers of cardiovascular disease in acute or chronic cardiometabolic disorders^7^. Furthermore, Experimental models of heart failure have confirmed the potential role of OPG in the adaptation of the myocardium to failure as they found a significant increase in mRNA expression of OPG in ischemic and non-ischemic myocardium with heart failure compared with that in subjects without heart failure^37^.

## Conclusion

TDT patients had lower T2* and EF than controls. Furthermore, OPG rs2073618 (G > C), RANK rs75404003 (C > DEL), and the A allele of RANKL rs2277438 (G > A) had significant relation with Myocardial iron overload and cardiac dysfunction occurring in TDT children. Therefore, the OPG/RANK/RANKL pathway impacts iron-overload-induced cardiac dysfunction in TDT children, and the related polymorphisms may act as genetic markers for iron-induced cardiomyopathy in these children.

### Study limitations

Assessment of the genetic status of the OPG/RANK/RANKL axis on a broader scale is recommended. In addition, other genes involved in the OPG/RANK/RANKL pathway should also be studied concerning their effect on iron-overload-induced cardiac dysfunction in transfusion-dependent thalassemia patients.

## Data Availability

The datasets used and/or analysed during the current study are available from the corresponding author on reasonable request.

## Abbreviations

TDT: Transfusion-dependent thalassemia
RANK: Receptor activator of nuclear factor-κB
RANKL: Receptor activator of nuclear factor-κB ligand
OPG: Osteoprotegerin
EF: Ejection fraction
CMRI: Cardiac MRI
